# Protein hydrolysis and fermentation under methanogenic and acidifying conditions

**DOI:** 10.1186/s13068-019-1592-7

**Published:** 2019-10-26

**Authors:** Thu Hang Duong, Katja Grolle, Tran Thi Viet Nga, Grietje Zeeman, Hardy Temmink, Miriam van Eekert

**Affiliations:** 1 Department of Environmental Technology, Wageningen University and Research, Bornse Weilanden 9, 6708 WG Wageningen, The Netherlands; 2 Faculty of Environmental Engineering, National University of Civil Engineering, 55 Giai Phong, Hanoi, Vietnam; 3 LeAF BV, PO Box 500, 6700 AM Wageningen, The Netherlands

**Keywords:** Proteins, Hydrolysis, Amino acid fermentation, Methanogenic conditions, Non-methanogenic conditions

## Abstract

**Background:**

Many kinds of wastewaters contain appreciable quantities of protein. Anaerobic processes are suitable for the treatment of wastewater high in organics to achieve pollution control and recovery of energy as methane and hydrogen, or intermediates for production of biofuels and valuable biochemicals. A distinction between protein hydrolysis and amino acid fermentation, especially for dissolved proteins, is needed to target which one is truly rate-limiting and to effectively harvest bioproducts during anaerobic conversion of these wastewaters. This study explored mesophilic anaerobic hydrolysis and amino acid fermentation of gelatine, as a model for dissolved proteins, at pH 7 and at pH 5.

**Results:**

The results showed that at pH 7, protein hydrolysis (first-order rate of 0.15 h^−1^) was approximately 5 times faster than acidification of the hydrolysis products (first-order rate of 0.03 h^−1^), implying that not hydrolysis but acidification was the rate-limiting step in anaerobic dissolved protein degradation. This was confirmed by (temporary) accumulation of amino acids. Nineteen different amino acids were detected during the first 8 incubation hours of gelatine at neutral pH and the total chemical oxygen demand (COD) of these 19 amino acids was up to approximately 40% of the COD of the gelatine that was added. Protein hydrolysis at pH 5 was 2–25 times slower than at pH 7. Shifting the initial pH from neutral to acidic conditions (pH 5) inhibited protein degradation and changed the volatile fatty acids (VFA) product profile. Furthermore, the presence or absence of methanogenic activity did not affect the rates of protein hydrolysis and acidification.

**Conclusions:**

The findings in this study can help to set a suitable solid retention time to accomplish anaerobic degradation of protein-rich wastewaters in continuous reactor systems. For example, if the target is harvesting VFAs, methanogens can be washed-out for a shorter retention time while amino acid fermentation, instead of hydrolysis as assumed previously, will govern the design and solutions to improve the system dealing with dissolved proteins.
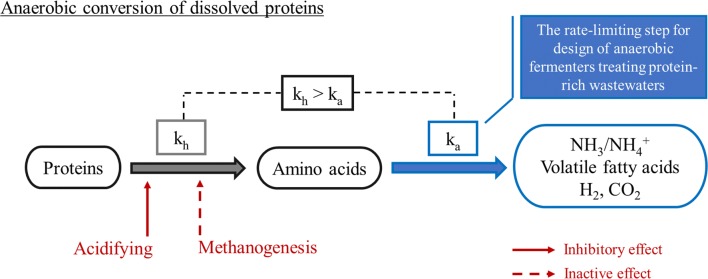

## Background

Anaerobic digestion is widely used for the treatment of high-strength wastewaters and organic wastes since it can combine pollution control with the recovery of methane or hydrogen as a green source of energy. Besides, volatile fatty acids (VFAs), important intermediates in anaerobic processes, recently have gained a lot of attention because they are platform chemicals for the production of more valuable compounds such as bio-based plastics, medium chain length fatty acids and other organic acids for bio-electrochemical systems [1–4]. Proteins are one of major compounds in wastewaters and wastes. Proteins account for 20–40% COD (chemical oxygen demand) in domestic wastewater, and up to 60–90% COD in sewage sludge and food wastewaters such as from the dairy, beverage, slaughterhouse and fish-processing industry [5–11]. Microorganisms cannot take up proteins directly but need extracellular proteases to cleave proteins in amino acids and small peptides, which can be subsequently taken up, metabolized to volatile fatty acids, ammonium and sulfide under acidogenic conditions and finally converted to methane under methanogenic [12]. Amino acids and peptides can also be used to synthesize cell proteinaceous matter, in particular when sufficient energy is present in the form of carbohydrates [13].

Anaerobic degradation of proteins is reported to be slower compared to degradation of other biopolymers [14–18]. For example, carbohydrates are considered to be favourable acidified than proteins in dairy wastewater [17]. Similarly, Khiewwijit et al. [19] observed that proteins were the main residual compounds after anaerobic treatment of domestic wastewater. Also, protein-containing wastewaters have been reported to result in low biogas yields, foaming and biomass wash-out and a deteriorated effluent quality [8, 20, 21].

Information about protein fermentation is insufficient. Proteins such as gelatine and casein were observed to be hydrolysed only to a minor extent under acidic conditions, either because of a reduced protease activity at low pH [14–17] or because of a lack of methanogenic activity under these conditions [22]. Sasaki et al. [23] observed that thermophilic acidification of protein (gelatine, casein, and bovine serum albumin) was enhanced by the presence of hydrogen-scavenging methanogens. Besides, presence or accumulation of intermediates as acetic acid during anaerobic degradation of gelatine could reduce gelatine hydrolysis rate in an anaerobic, mesophilic saline environment [24].

Most studies concluded that protein hydrolysis is the rate-liming step while subsequent amino acid fermentation was fast [25–27]. However, this conclusion may be questionable because it was based on ammonium release rates, which does not allow for a distinction between protein hydrolysis and amino acid fermentation [25, 26].

Free amino acids and peptide concentrations were rarely measured in studies focussing on anaerobic treatment of protein-rich wastewater. Although Breure and Van Andel [14] and Miron et al. [22] mentioned the presence of amino acids during protein degradation, they did not sufficiently quantify their concentrations to be able to compare hydrolysis and acidification rates. More knowledge is available from protein degradation in the rumen. Broderick et al. [28] observed accumulation of peptides and amino acids within the first 2 h after feeding ruminal bacteria with silages. Later, Cardozo et al. [29] found in continuous fermenters receiving a daily diet of forage considerable concentrations of peptides and amino acids after 8 h feeding. These findings indicate that amino acid fermentation or deamination could be the rate-liming step during anaerobic protein degradation.

In the present study we explored the hydrolysis and degradation of gelatine as a model dissolved protein under methanogenic and non-methanogenic conditions at a neutral pH, and at a low pH of 5. For this purpose batch experiments were employed with an inoculum taken from a continuous fermenter that was fed with milk to represent a microbial population adapted to wastewater from the dairy industry. Protein degradation was followed in time, not only based on COD concentrations and gas production, but also the protein concentration and the amino acid and VFA concentration and composition.

## Results and discussion

### Effect of methanogenic conditions on gelatine degradation

**Fig. 1:**
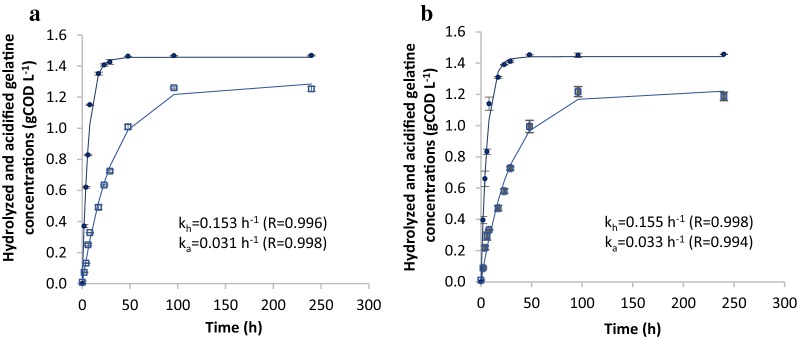
First-order model for hydrolyzed and acidified gelatine concentrations in gelatine-pH7 (**a**) and gelatine-BES-pH7 (**b**) in the batch experiment at 35 °C. (Data plotted the mean and standard deviation). Decrease of protein (filled circle), VFA and CH_4_ (open square), first-order model (continuous line)

Previous research indicated that methanogenesis stimulates anaerobic protein degradation [22–24]. Figure 1 shows the hydrolyzed and acidified gelatine concentrations at pH 7 (left) and at pH 7 where methanogenesis was inhibited with 2-bromoethanesulfonate (BES), (right), which was confirmed by a lack of methane production. In both cases hydrolysis as well as acidification could be described by the first-order kinetics of Eqs. (1) and (2), see in “Calculations” section. Interestingly, in contrast to the literature the occurrence of methanogenesis did not affect the hydrolysis and acidification rate. This observation was confirmed from amino acid measurements, as will be explained in “Amino acid production and fermentation” section.

More than 99% of the initial 1.40 g COD L^−1^ of (dissolved) gelatine was hydrolysed during the experiments, whereas 1.25 and 1.20 g COD L^−1^ of acidification products were measured under methanogenic and non-methanogenic conditions, respectively. This difference between the extent of hydrolysis and acidification can be explained by part of the COD being used for biomass production (see “COD mass balance” section for more details).

The first-order rate constant for protein hydrolysis (*k*_h_) (0.153 and 0.155 h^−1^ under methanogenic and non-methanogenic conditions, respectively) were much higher than the first-order rate constants of acidification (*k*_a_) (0.031 and 0.033 h^−1^). This implies that not protein hydrolysis but acidification of the hydrolysis products was the rate-limiting step for anaerobic dissolved protein degradation.

Sanders et al. [30] showed that the dissolved protein (gelatine) hydrolysis rate was related to sludge concentration and to gelatine concentration. In their tests, comparable to ours, the gelatine hydrolysis rate was modelled using a zero-order kinetics model during the initial incubation hours. We observed an initial hydrolysis rate of 0.137 and of 0.140 g COD L^−1^ h^−1^ during the first 8 h of the batch tests, which is in accordance with the results of Sanders et al. [30] (0.15 g COD L^−1^ h^−1^) at a similar VS-sludge inoculum to COD-gelatine concentration ratio of 5 g volatile solids (VS) g^−1^ COD.

The first-order hydrolysis rate constants of 0.153–0.155 h^−1^ are higher than those reported by others [14, 31]. This may be explained by the calculation of the hydrolysis rate constant based on VFA production [32] and/or ammonium production [25, 33], which may have underestimated the hydrolysis rate. However, it is appreciated that many other factors such as the type of protein and biomass inoculum [26, 31], the biomass to protein ratio [30] and temperature [25] may also explain the differences.

### Effect of low pH on gelatine hydrolysis

The pH variation in the Gelatine and Gelatine-BES at pH 7 during the experiment was negligible. The pH in Gelatine-pH 5 bottles increased to pH 5.5 during the first 48 h, but then stabilized at this value.

**Fig. 2:**
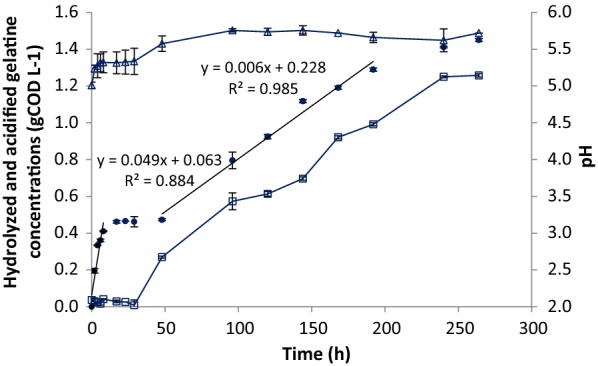
The depletion of gelatine concentration and hydrolysis rate at different periods, change of acidified gelatine concentrations and pH during incubation time at gelatine-pH 5 in the batch experiment at 35 °C. (Data plotted the mean and standard deviation). Decrease of protein (filled circle), VFAs and CH_4_ (open square) and pH (open triangle)

Figure 2 shows the concentration of hydrolyzed gelatine in the bottles at pH 5 together with VFA production and pH. Nearly no methane was formed. After an initial hydrolysis rate of approximately 0.05 g COD L^−1^ h^−1^ gelatine degradation stagnated between 8 and 48 h. After 48 h, gelatine hydrolysis took off again, albeit at a much lower rate of 0.006 g COD L^−1^ h^−1^. These rates are 2–25 times lower than the initial hydrolysis rate of 0.137 and of 0.140 g COD L^−1^ h^−1^ observed in the experiments at pH 7, under methanogenic and non-methanogenic conditions, respectively. Breure and Van Andel [14] found in a chemostat system at 30 °C a gelatine hydrolysis rate at pH 5 that was twice as low as at pH 7.

The hydrolysis of gelatine at pH 5 during the first 6–8 h was probably related to the presence of proteolytic enzymes in the inoculum which, to a certain extent, was still active. The inhibition of gelatine hydrolysis between 8 and 48 h may be related to a negative effect of a low pH on the activity of the hydrolytic enzymes. A similar effect was observed by Lu et al. [34] with a low protease activity under acidic conditions when starting up anaerobic digestion of municipal solid waste. Breure and Van Andel [14] reported an optimum pH of proteases of 7.5 whereas at a pH of 5 this was reduced by 50%. This negative effect may be due to an electrostatic repulsion among charged active sites of the proteolytic structure at pH 5. Similarly, at low pH, attachment of the gelatine to cell bound proteases becomes more difficult as has been reported for ruminal organisms [26, 28]. Finally, at low pH the fraction of undissociated VFA is higher, which has a negative effect on microbial growth [35–37] and herewith perhaps also on the excretion of proteolytic enzymes. To our knowledge, an effective hydrolysis of proteins at acidifying conditions has not been reported yet in literature, although adaptation to a low pH cannot be excluded [16, 20].

### Amino acid production and fermentation

**Fig. 3:**
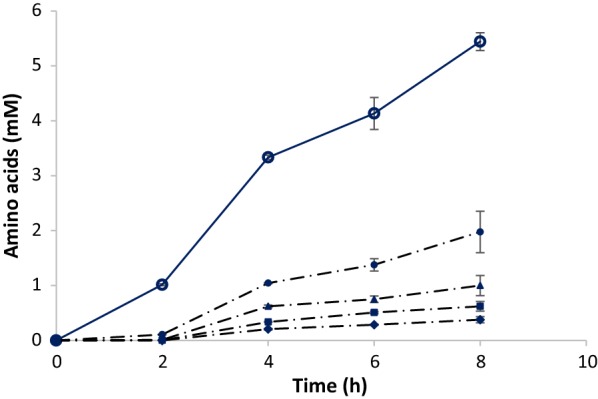
The concentration of total amino acids and of glycine, alanine, proline and glutamic acid during the first 8 h of anaerobic degradation of gelatine at pH 7, at 35 °C. (Data plotted the mean and standard deviation). Total amino acids (mM) (open circle), glycine (filled circle), alanine (filled triangle), proline (open square), and glutamic acid (filled diamond)

In the Gelatine (Fig. 3) and Gelatine-BES bottles, nineteen different amino acids were detected during the first 8 h of incubation, at concentrations up to 2 mM. Glycine, alanine, proline and glutamic acid were detected at the highest concentrations. The total COD of these 19 amino acids was as high as 0.60 g COD L^−1^ in the Gelatine bottles and 0.63 g COD L^−1^ in the Gelatine-BES bottles, which is equivalent to approximately 40% of the COD of the gelatine that was added. From 8 h onwards only very small amounts (0.01–0.1 mM) of amino acids were detected, and apparently these were readily fermented to VFAs. The temporary accumulation of amino acids at pH 7 under methanogenic and non-methanogenic conditions confirms that the initial hydrolysis rate of gelatine was much faster than the amino acid fermentation rate. Because amino acid accumulation was similar under methanogenic and non-methanogenic conditions this confirms that methanogenic conditions are not a prerequisite to obtain fast protein hydrolysis. So far, concentrations of different amino acids have not been measured or reported for anaerobic degradation of protein-rich (waste)waters. However, in studies with ruminal microorganisms accumulation of free amino acids during degradation of food-containing proteins was observed by Broderick et al. [28] and Cardozo et al. [29]. Clearly, our results imply that amino acid fermentation can be the rate-limiting factor for protein degradation not only in the rumen [13] but also in anaerobic wastewater treatment reactors. Therefore, this should be taken into account when designing such reactors (also see “Consequences for design and operation of anaerobic reactors for protein-rich wastewaters” section).

In the pH 5 bottles after 8 h only 0.01–0.2 mM of amino acids were detected, equivalent to a total of 0.07 g COD L^−1^. This agrees with the observation made earlier that hydrolysis of gelatine into amino acids at this low pH was very slow. Apparently, at pH 5, acidification was not the rate-limiting step.

**Fig. 4:**
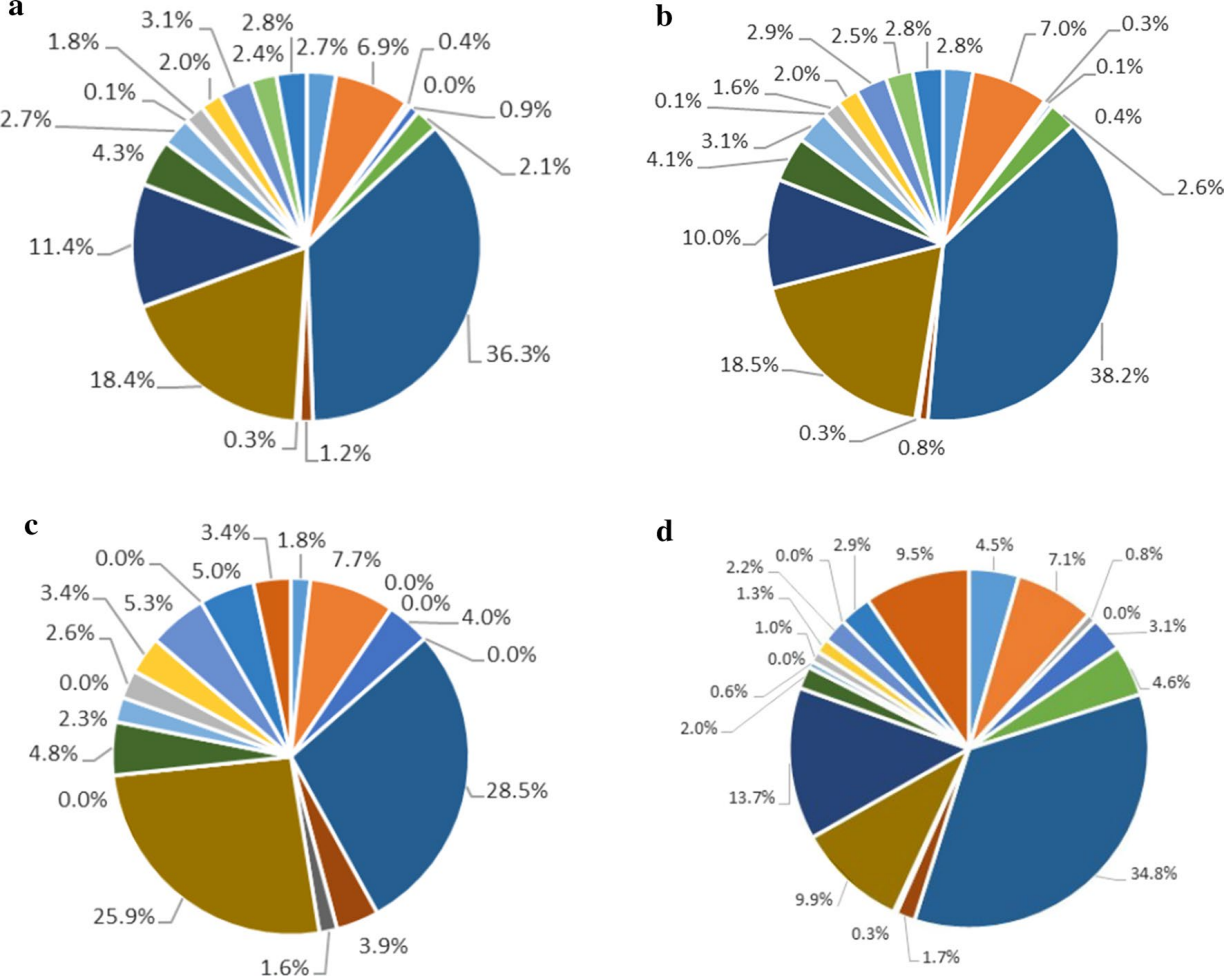
Percentage of amino acids (in total mM) from gelatine-pH7 (**a**), gelatine-BES-pH7 (**b**), and gelatine-pH 5 (**c**) after 8 h of incubation at 35 °C; Data expressed as the mean value (*n* = 3, standard deviation less than 5%). Theoretical amino acids composition of gelatine (referenced from Gelatin handbook, 2012) shown in **d**. Aspartic acid (  ), arginine (  ), proline (  ), phenylalanine (  ), glutamic acid (  ), glycine (  ), valine (  ), leucine (  ), histidine (  ), threonine (  ), methionine (  ), cystine (  ), glutamine (  ), tyrosine (  ), tryptophan (  ), lysine (  ), serine (  ), alanine (  ), isoleucine (  ), hydroproline (  )

Figure 4 shows the amino acids detected during 8 h of incubation time under the different conditions along with the “theoretical” composition of gelatine [38]. The amino acid composition at pH 7 was similar to the amino acid composition of gelatine, except for a slightly higher percentage of alanine by 8% and the absence of hydroproline. This indicates that gelatine hydrolysis was rather unselective. At pH 5, a lower contribution of glycine (by 7%) and a higher contribution of alanine (by 16%) were detected after 8 h of incubation. Apparently at both pHs alanine was more slowly acidified to VFAs than the other amino acids.

### VFA production

**Fig. 5:**
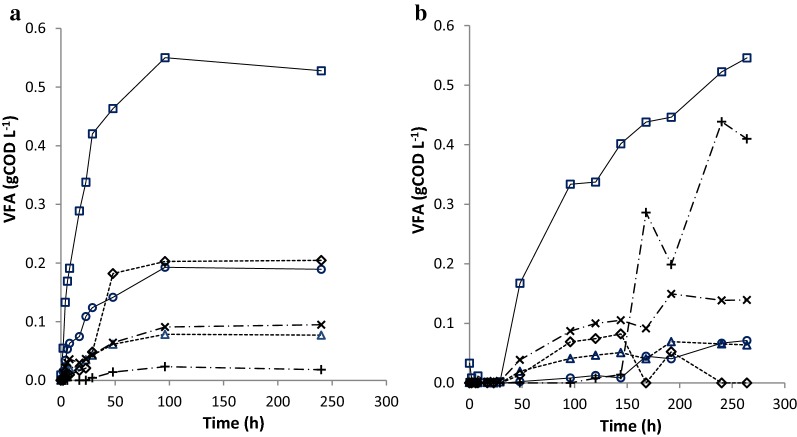
Concentration of VFAs from gelatine degradation at pH7 (**a**) versus at pH5 (**b**) in the batch experiment at 35 °C. Data expressed as mean value (*n* = 3, with standard deviation less than 5%). Acetate (open square), propionate (open circle), i-butyrate (open triangle), *n*-butyrate (open diamond), i-valerate (x), *n*-valerate (+)

Figure 5 shows the VFA produced during gelatine degradation at pH 7 under non-methanogenic conditions (left) and at pH 5 (right). Because methanogenesis was absent, in both cases VFA accumulated, with acetate accounting for 45% of the total VFA.

At pH 7, VFA was rapidly produced during the first 50 h of incubation. Acetate reached a concentration of 0.55 g COD L^−1^ and the total VFA concentration was 1.14 g COD L^−1^, equivalent to 83% of the gelatine-COD that was added. At pH 5 almost no VFA was detected until after 29–48 h of incubation. This slower production of VFA at pH 5 compared to pH 7 was already explained above as hydrolysis of gelatine into amino acids at pH 5 was lower than at pH 7. Another difference between pH 5 and pH 7 was that at pH 5 *n*-valerate was the second most abundant VFA that was produced while it was produced the least at pH 7, showing that the VFA spectrum is determined by the pH [4]. The VFA profiles are partly in accordance with [4, 14, 39] who observed that shifting pH from 7.0 to 5.0 gradually decreased the production of acetate and butyrate and promoted the production of (*n*-)valerate from proteins. This can be explained by a lower energy expenditure to excrete larger VFAs compared to smaller molecules. As a result, at lower pH valerate production is more favourable.

### COD mass balance

**Fig. 6:**
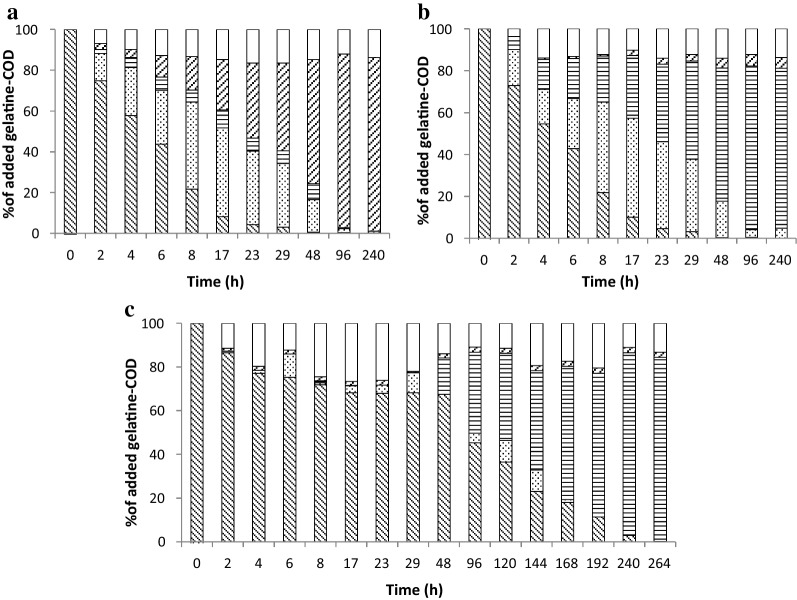
Anaerobic conversion of gelatine-COD to amino acids and peptides-, VFAs- and CH_4_-COD in gelatine-pH 7 (**a**), gelatine-BES-pH 7 (**b**) and gelatine-pH 5 (**c**) in the anaerobic batch experiment at 35 °C. Data expressed as mean value (*n* = 3, with standard deviation less than 5%). Gelatine (  ), amino acids and peptides (  ), VFAs (  ), CH_4_ (  ), missing-COD (open square)

Figure 6 shows mass balances for the different batch experiments. Gelatine was completely (> 99%) converted at the end of the batch tests and the final products (methane and VFA) accounted for 83–86% of the COD, regardless of the pH and methanogenic activity. The missing 14–17% of the COD can be attributed to biomass production and is in a range of biomass yield of 0.12–0.36 g COD g^−1^ protein-COD reported by others [12, 14, 17, 25, 27].

### Consequences for the design and operation of anaerobic reactors for protein-rich wastewaters

The results of this study clearly showed that not hydrolysis but subsequent acidification of the hydrolysis products is the rate-limiting step in anaerobic conversion of dissolved proteins. Obviously this has consequences for the design of anaerobic treatment reactors. At pH 7 a hydrolysis rate constant of 0.15 h^−1^ and an acidification rate of 0.03 h^−1^ were found for gelatine. As an example, to avoid amino acid accumulation in a Completely Stirred Tank Reactor (CSTR) the volume of this CSTR should be 5 times bigger than the volume in case only protein hydrolysis would be taken into account. It is strongly recommended that the above difference between the rate of hydrolysis and acidification of the amino acids is also considered in models such as the anaerobic digestion model No. 1 that are used for design purposes [40].

Interestingly, the large difference between the hydrolysis and acidification rates offers the possibility to avoid the degradation of amino acid and design the reactor such that they can be recovered. For instance, valine, leucine and iso-leucine are important substrates for branched fatty acids formation such as iso-butyrate, iso-valerate and iso-caproate that can be harvested from fermenting protein-rich waste streams for potential branched chain elongation [41].

The production of VFA from waste streams has gained a lot of attention because they are considered important intermediates for the production of higher value products such as bioplastics. To this end reactors are operated at a short solids retention time to wash-out methanogens that otherwise would consume the VFAs. If the waste stream also contains an appreciable amount of (dissolved) proteins a low pH should be avoided at all times because it was shown that at pH 5 protein hydrolysis is approximately 20 times slower than at pH 7. Not only would this result in a lower VFA yield, but also in problems associated with the presence of proteins such as foaming, biomass wash-out and a deteriorated effluent quality [8, 20, 21]. Adaptation or acclimation could potentially enhance protein hydrolysis at low pH [20, 42], but this requires more research in this direction.

## Conclusions

Batch experiments carried out with gelatine under mesophilic conditions (35 °C) showed that the hydrolysis rate constant of gelatine was faster than the acidification rate, leading to accumulation of free amino acids. It is concluded that not hydrolysis but acidification can limit the fermentation rate of dissolved proteins. Methane formation did not stimulate the protein hydrolysis and acidification at neutral pH. Shifting the initial pH from neutral to acidic conditions (pH 5) inhibited protein degradation and changed the VFA product profile. The findings in this study can be used to define retention times needed for efficient anaerobic treatment of protein-rich wastewaters.

## Methods

### Experimental set-up

#### Substrate

The model protein was gelatine, CAS no. 9000-70-8 (Merck, for microbiology, 1.04070.0500). The gelatine was completely dissolved in heated demi-water (40–50 °C). The pH of the gelatine solution was 5.0–5.5 and the concentration of gelatine below 2% to ensure a random coil configuration of gelatine and negligible electrostatic disturbance that might change the protein structure [43]. It was shown by others that the pH in the experimental range of 5–7 did not influence gelatine structure and solubility [44]. The main characteristics of the gelatine are shown in Table 1.

**Table 1:** Main characteristics of the protein used in this experiment

Characteristics	g TS	g VS	g COD	g TN
Protein (gelatine)	0.953 ± 0.004	0.952 ± 0.004	1.150 ± 0.013	0.139 ± 0.001

Data are measured per gram gelatine and expressed in mean ± standard (*n* = 10)

#### Inoculum and nutrient medium

The seed sludge for the batch tests was harvested after an operational period of 150 days from a continuous fermenter that was operated at a volumetric loading rate of 2 g COD L^−1^ d^−1^ and was fed with fresh milk. The fermenter had a working volume of 10 L (total volume 14 L), and was operated at 35 °C. The pH in the reactor was 7.3. The total solids (TS) and volatile solids (VS) of the sludge were 19 g L^−1^ and 12 g L^−1^, respectively. The nutrient medium for the batch tests was adapted from Angelidaki et al. [45] except that NH_4_Cl was not added because sufficient nitrogen was already present in the sludge inoculum [total nitrogen (TN) of 3.8 g L^−1^ and NH_4_-N of 3.6 g L^−1^]. Each liter of the nutrient medium at pH 7 contained 2.18 g Na_2_HPO_4_; 1.06 g KH_2_PO_4_; 48 mg CaCl_2_·2H_2_O; 54 mg MgSO_4_·7H_2_O; 1.2 mg FeCl_2_·4H_2_O; 1.2 mg CoCl_2_·6H_2_O; 0.3 mg MnCl_2_·4H_2_O; 0.018 mg CuCl_2_·2H_2_O; 0.03 mg ZnCl_2_; 0.03 mg HBO_3_; 0.054 mg (NH_4_)_6_Mo_7_O_24_·4H_2_O; 0.06 mg Na_2_SeO_3_·5H_2_O; 0.03 mg NiCl_2_·6H_2_O; 0.6 mg EDTA (tripex II); 0.216 mL HCl 36%; 0.3 mg Resazurin. The medium at pH 5 was prepared to be identical to the medium at pH 7, except KH_2_PO_4_ (3.128 g L^−1^) and none of Na_2_HPO_4_.

#### Anaerobic batch experiments

The experiments were carried out in triplicate at 35 °C in 2.6 L side-port-bottles (liquid volume of 0.62 L), which were continuously shaken at 60 rpm for 300 h. Three different sets of test bottles were prepared: (i) gelatine bottles at pH 7 with a gelatine concentration of 1.46 (± 0.015) g COD L^−1^ and an inoculum of 7.0 (± 0.05) g VS L^−1^ (non-adjusted pH of the culture); (ii) gelatine-BES bottles similar to the protein pH 7 bottles but with addition of 2-bromoethanesulfonate sodium (BES, 0.02 M) to inhibit growth of methanogens; (iii) gelatine-pH 5 bottles similar to the Gelatine-pH 7 bottles with addition of hydrochloric acid (HCl, 0.075 M) to obtain a pH of 5. Blanks were prepared with seed sludge and medium but without gelatine. Before they were closed all the bottles were well-mixed, sampled for initial concentrations and flushed with N_2_ gas for 30 min.

An additional test was conducted with (i) gelatine and (ii) gelatine-BES (pH 7) amended with NaCl (± 0.075 M) to verify that chloride (Cl^−^) at this concentration in gelatine-pH 5 did not have a negative effect on gelatine hydrolysis and degradation.

### Sampling and analyses

During the first 8 h gas and liquid samples were taken from the bottles with an interval of 2 h. Afterwards, six more samples were taken from all bottles at 17, 23, 29, 48, 96, and 240 h after start of the incubation. Five additional samples were taken from gelatine-pH 5 bottles after 120, 144, 168, 192, and 264 h.

pH was measured by a pH meter (Hach, PHC 101, Seri No.162822568077, USA). The sludge samples were centrifuged (Eppendorf, Germany) at 10000 rpm for 10 min and filtered with pre-washed 0.45 µm cellulose acetate membrane filters (Sartorius, Germany). The soluble fraction was analyzed for chemical oxygen demand (CODs), total nitrogen (TN) and ammonium (NH_4_-N) using Hach Lange methods and test kits (LCK1014, LCK338, LCK303). Protein was determined using the Lowry method assay [46] at 660 nm using gelatine as standard. Volatile fatty acids (VFAs) were quantified on a Trace gas chromatograph equipped with a Thermo TR-WAX column (30 m × ID 0.32 mm × thickness of 0.25 µm) connected to a FID detector as described by Sudmalis et al. [47]. Amino acids were measured in the supernatant samples as described by Meussen et al. [48] via high-performance liquid chromatography (HPLC). Carbohydrate was determined by the phenol–sulfuric acid method [49] at 490 nm using glucose as standard. Carbohydrate concentrations were only measured at 0, 17, 48 and 240 incubation hours from all the bottles to verify the negligible effect of the presence of carbohydrate. It was confirmed that carbohydrate did not have an effect on protein hydrolysis and cell synthesis because the carbohydrate concentrations in all the bottles were identical at 0.04–0.05 g COD L^−1^ and did not change over the time.

Gas pressure in the head space, as a measure for biogas production, was determined by TSI Certifier FA Plus (USA, model 4088A, SN 40880735005). Gas composition (CH_4_, CO_2_, H_2_ and N_2_) was quantified by gas chromatography-8A (Shimadzu, Japan) equipped with a compact materials Unibeads C 60/80 mesh column (*Φ*3 mm, length 2 m) connected to a thermal conductivity detector (argon as carrier gas). Sludge inoculum and gelatine substrate solution of the batch tests were analysed for TS and VS using standard methods [50].

### Calculations

The rate of protein hydrolysis depends on the sludge concentration. However, in the batch tests a constant sludge concentration was applied. Therefore, to be able to compare hydrolysis rates first-order hydrolysis kinetics were assumed as proposed by Batstone et al. [40] and the concentration of hydrolyzed protein in time was described by:

1 $$P_{\text{hydrolyzed}} \left( t \right) = P_{\text{added}} .\left( {1 - \exp \left( { - k_{\text{h}} .t} \right)} \right)$$

With $P_{\text{hydrolyzed}} \left( t \right)$ the (cumulative) concentration of hydrolyzed protein (g COD L^−1^) after *t* hours, $P_{\text{added}}$ the initial concentration of protein (g COD L^−1^) and $k_{\text{h}}$ the first-order hydrolysis rate constant (h^−1^). The protein concentration was calculated as COD from the measured soluble protein concentration using a conversion factor of 1.115 g COD g^−1^ gelatine (Table 1).

Subsequent acidification of the hydrolysis products was also described by first-order kinetics according to:

2 $$P_{\text{acidified}} \left( t \right) = P_{\text{end-acidified}} .\left( {1 - \exp \left( { - k_{a} .t} \right)} \right)$$

where $P_{\text{acidified}} \left( t \right)$ is the sum of the measured VFA concentration and methane production after *t* hours, both expressed in g COD L^−1^, $P_{\text{end-acidified}}$ is the sum of the measured VFA concentration and methane production at the end of the tests (in g COD L^−1^) and $k_{\text{a}}$ is the first-order acidification rate constant (h^−1^).

## Data Availability

All data generated or analysed during this study are included in this published article.

## Abbreviations

BES: 2-Bromoethanesulfonate
COD: Chemical oxygen demand
TN: Total nitrogen
TS: Total solids
VFA: Volatile fatty acid
VS: Volatile solids
